# Recent Progress in Discovering the Role of Carotenoids and Metabolites in Prostatic Physiology and Pathology—A Review—Part II: Carotenoids in the Human Studies

**DOI:** 10.3390/antiox10020319

**Published:** 2021-02-20

**Authors:** Joanna Dulińska-Litewka, Przemysław Hałubiec, Agnieszka Łazarczyk, Oskar Szafrański, Yoav Sharoni, James A. McCubrey, Bartosz Gąsiorkiewicz, Torsten Bohn

**Affiliations:** 1 Chair of Medical Biochemistry Medical College, Jagiellonian University, 31-034 Cracow, Poland; przemyslawhalubiec@gmail.com (P.H.); agnieszka.lazarczyk@student.uj.edu.pl (A.Ł.); osk.sza2@gmail.com (O.S.); b.gasiorkiewicz@student.uj.edu.pl (B.G.); 2 Department of Clinical Biochemistry, Faculty of Health Sciences, Ben-Gurion University of the Negev, P.O. Box 653 Beer Sheva, Israel; yoav@bgu.ac.il; 3 Department of Microbiology and Immunology, Brody Medical Sciences Building, East Carolina University, Greenville, NC 27834, USA; mccubreyj@ecu.edu; 4 Nutrition and Health Research Group 1 A-B, Population Health Department, Luxembourg Institute of Health, rue Thomas Edison, L-23 1445 Strassen, Luxembourg; Torsten.bohn@gmx.ch

**Keywords:** beta-carotene, lycopene, metabolism, apo-carotenoids, vitamin A, prostate cancer, antioxidants, cohort studies

## Abstract

Among the vast variety of plant-derived phytochemicals, the group of carotenoids has continuously been investigated in order to optimize their potential application in the area of dietary intervention related to chronic diseases. One organ that has been especially targeted in many of these studies and clinical trials is the human prostate. Without doubt, carotenoids (and their endogenous derivatives—retinoids and apo-carotenoids) are involved in a plethora of intra- and intercellular signaling, cell growth, and differentiation of prostate tissue. Due to the accumulation of new data on the role of different carotenoids, such as lycopene (LYC) and β-carotene (BC), in prostatic physiology and pathology, the present review aimed to cover the past ten years of research in this regard. Data from experimental studies are presented in the first part of the review, while epidemiological studies are disclosed in this second part. The objective of this compilation was to emphasize the present state of knowledge about the most potent molecular targets of carotenoids, as well as to propose promising carotenoid agents for the prevention and possible treatment of prostatic diseases.

## 1. Introduction

In the first part of the review [1], we covered the main directions of current investigations targeting the molecular basis of carotenoid actions, summarizing the results of ongoing research in the field.

This second part of the review is focused on the latest research in the field of associations of carotenoid intake and/or circulatory concentrations related to the incidence of malignancy of the most important pathology of prostate—prostate cancer (PC). Today, PC is the most abundant cancer in men worldwide [2]. In 2018, there were 1,276,106 new cases diagnosed around the globe, which accounted for 7.1% of total cancer incidence in men. Consistently, PC was the second most common cancer in American males, constituting 9.5% of all new cases in 2018 [3]. It is still being discussed whether such a high prevalence of PC is a result of an accumulation of risk factors (especially ageing of society, lack of physical activity, or improper diet), or whether it is the result of more widely carried out screening, including for a prostate-specific antigen (PSA). Baade et al. suggested that lower incidence of PC in certain developed countries could be explained by lesser popularity of PSA testing, such as in Japan or Poland [4]. On the other hand, significant differences in the incidence of PC were noticed according to the ethnicity, for instance 40-fold higher odds for developing PC among African-American men living in the United States compared to Asian men living in their native countries [2]. However, the aforementioned risk of PC tends to be increased in Asian men who migrated to ‘high-risk’ countries.

Presently, the average age of diagnosis of PC is 66 years and the general belief is that below the age of 50 years its incidence dramatically drops, with only 1 in 350 younger males being affected by the disease [3].

Since the previous decade, it has been hypothesized that carotenoids (particularly lycopene (LYC)) might exert a positive influence on the clinical outcomes of PC prophylaxis and treatment. Although LYC has been shown to improve the incidence and prognosis in humans and animals regarding gastric, breast, pancreatic, colon, and renal cancer, there have also been contrasting results reported in this area of research. Additionally, there is a scarcity of high quality observational and interventional studies performed in order to unequivocally establish the role of LYC in cancer disease [5,6]. In terms of PC, in 2004, Etminan et al. conducted a meta-analysis and concluded that adequate intake of tomato products (i.e., 200 g of tomatoes per day) could visibly reduce the incidence of the disease [7]. Unfortunately, further investigation has not brought any clear conclusion in this regard and, therefore, it remains one of goals of ongoing research to deliver the proper answer. It is, however, clear that LYC, among other carotenoids, is a highly bioactive compound with a multidimensional beneficial role in human health, which has been extensively reviewed [8].

In fact, it is reported to possess the strongest antioxidant properties among all the carotenoids, which is accomplished by both its ability to directly scavenge reactive oxygen species (ROS) and to upregulate the antioxidant response elements (ARE) [9]. Therefore, LYC is capable of reducing tumor cell proliferation, regulating the cell cycle, or inducing apoptosis of cancer cells [1,9]. LYC seems to exert beneficial effects also in the other fields of health, especially by acting as a cardioprotective and neuroprotective factor, and by maintaining skin resistance to photodamage [10].

The main aspects of carotenoids’ physiology and molecular mechanisms of action have been summarized graphically [1].

The fundamental issue that has hampered previous attempts to define the precise role of various factors related to PC progression (not only carotenoids) is the fact that the exact biology of PC itself is still under investigation. In 2015, the team setting up the The Cancer Genome Atlas proposed a new division of PC into seven molecular subtypes, differing in their androgen receptor (AR) activity in a subtype-specific manner [11]. In this part of the review, we try to emphasize our present knowledge of the role of carotenoids in prostate cancer and prostate-related disease, based on epidemiological studies in humans.

## 2. Materials and Methods

Here, we provide a brief description of the methodology. A full description of the methodology was given in part I [1].

### 2.1. Search Strategy and Study Selection

We have investigated electronic databases (PubMed, Cochrane, Ovid, NICE) in order to find relevant studies for our literature review. Studies considered included both intervention trials and observational trials determining the association between any carotenoids and prostate complications. Investigated populations included elderly men with prostate abnormalities (predominantly PC). For interventional studies, the independent variable was the amount of supplemented carotenoids or their serum concentration after intervention. Outcomes were assessed as the presence of disease—for PC, measured mainly by screening tests, PSA, and digital rectal examination (DRE). We decided to extract data from papers published between the 1st of January 2009 and the 15th of November 2020.

All received results were manually checked by two authors. The main eligibility criteria were: studies investigating the impact of any carotenoid on aspects of prostate physiology and/or pathology.

### 2.2. Data Extraction

Two authors were chosen to independently find studies, which met the inclusion criteria. If there were any conflicting situations, a third author was engaged for a final decision.

### 2.3. Presentation of Results

An analysis of epidemiological studies was done following the Meta-analysis of Observational Studies in Epidemiology (MOOSE) standard [12]. However, due to the strong heterogeneity in their methodology and approaches (as discussed in the following), we decided that none of the statistical models for meta-analyses would result in appropriate conclusions and, therefore, we relied on a discussion of unprocessed data from these studies. To estimate the methodological quality of studies, the Newcastle–Ottawa Scale was used [13].

The flow chart summarizing the process of data extraction is presented as Figure S1 in Online Supplementary Material.

## 3. Carotenoids and Prostate Cancer in Epidemiological Studies

### 3.1. Carotenoids and Prostate Cancer Risk—Introduction

We identified 23 research articles referring directly to the influence of carotenoids on PC occurrence. All but one were performed with human subjects. The predominant investigated carotenoids were LYC (n = 14) and β-carotene (BC) (n = 13). In addition, articles contained results in relation to retinol (or retinyl esters), α-carotene, β-cryptoxanthin, zeaxanthin/lutein, phytoene, and phytofluene. Regarding the type of study and its design, there were 2 controlled clinical trials, 6 cohort studies, 11 case-control studies, and 3 cross-sectional studies. The largest one was the Health Professionals Follow-up Study, which presented 5728 PC cases. Basic information about each study (except for the one animal study) is shown in Table 1. A lot of heterogeneity existed among the results. Four studies with significant results concluded that carotenoids even increased the risk of PC. Before taking a closer look at particular studies, we discuss basic factors that appeared to influence the outcome of the investigations.

Due to the qualitative nature of the analysis, the assessment of risk of bias was done adhering to the GRADE-CERQual. Following this step, we focused on methodological limitations, coherence, adequacy of data, and relevance of included studies [14].

1. Number of cases—eight studies included less than 100 cases of PC [15,16,17,18,19,20,21,22] and eight more than 1000 cases [23,24,25,26,27,28,29,30]. In fact, mainly studies with <100 cases reached statistically significant results. For example, Karppi et al. [15] found in their study that both α-carotene (AC) and BC significantly increased the risk of PC by about 105% and 129%, respectively (comparing the highest vs. the lowest tertiles of concentrations). Contrarily, Nordström et al. [20] stated a 69% decrease of PC risk (however there were no particular concentrations of carotenoids specified, which makes the results of this study incomparable). These examples demonstrate that especially small-scale studies may be more prone to diverging outcomes, due to differences in the nature of the study design, populations included, carotenoid administration schemes, length of intervention, and many more.
2. Nationality of participants—the nationalities that have been most frequently investigated are: American (n = 6), Finnish (n = 5), and Italian (n = 2). There were also Australian, Japanese, and Vietnamese populations. Four studies included multiethnic groups of participants. The prevalence of PC strongly differed, depending on the geographic region. According to the Global Cancer Statistics, in northern America, PC incidence is 73.7 (out of 100,000), 85.7 in northern Europe, 60.7 in southern Europe, 86.4 in Australia, 13.9 in eastern Asia, 12.7 in southeastern Asia, and 64.1 in southern Africa (age-standardized rate per 100,000) [31]. It is possible that the susceptibility to carotenoids depends on specific configurations of genetic polymorphisms, but influences regarding lifespan, pollution, and other factors, such as sun exposure etc., may also likely play a role. Moreover, such associations between genetics and environmental factors have been already suggested and shown (e.g., for TMPRSS2: ERG) [22]. Other factors confounding the distribution of PC are access to medical care, physical activity, dietary habits, and addictions—factors that are also strongly related to the country of investigation. For example, in a Vietnamese study by Van Hoang et al. [32], which took place from 2013 to 2015, it was suggested that results should be interpreted carefully for the Vietnam population, as only the Ho Chi Minh City population was investigated. Accordingly, conclusions should be rather restricted to the investigated populations instead of trying to generalize them.
3. Evaluation of outcome—this is probably the most confusing factor explaining the high level of inconsistency among results of different studies. First, none of the prospective studies could guarantee that the outcome—PC development—was not present at the beginning of study. For example, the two included controlled clinical trials [23,33] extracted data about diagnosis from corresponding cancer registries. However, this solution has two great disadvantages. Diagnosis of PC is indicated by raised PSA levels or pathological findings in DREs. Lower urinary tract symptoms could lead to diagnosis as well. If there are none of these indications, PC would remain undiscovered. None of the studies began by a diagnostic biopsy to exclude the presence of PC before an intervention. It is understood that starting research with invasive procedures is a large technical and ethical difficulty and could not be achieved—it should be merely stressed that among people aged 50–69 years (the dominating group in the conducted studies), there is a significant chance to find clinically silent, developing PC. The mean age of PC diagnosis in the US is 66, but average time of tumor growth is estimated as 10 years [2].

In turn, there is one important example of how post-research evaluation is likewise important, as presented by Kristal et al. [26]. During their Prostate Cancer Prevention Trial (PCPT) study, which lasted for nine years, 772 cases of PC were found. At the end of the study, all participants underwent a “not-for cause biopsy” (while “for cause biopsy” means one indicated by PSA or DRE screening, or lower urinary tract symptoms). From this, 911 cases of PC were revealed, which constituted 54.1% of all cases. This strongly suggested that underdiagnosing of PC could contribute to a large diagnostic bias (there has been no other study that introduced the idea of a “not-for cause biopsy”). As the outcome assessment of all studies might have been altered by such additional measurements, we should pay particular attention to the borderline results (both positive and negative).

4. Evaluation of carotenoids—there were three main methods used for calculating a relationship between PC incidence and carotenoids: (a) patients were given supplements containing a known quantity of carotenoids [18,23]; (b) carotenoid concentration in serum was measured [15,16,17,19,20,25,26,27,28,30,33,34]; and (c) carotenoid intake was estimated according to the results of standardized food frequency questionnaires [21,22,24,29,32,35]. Serum concentrations of carotenoids are considered to be correlated with PC in a more direct way than dietary intake [26], despite both being correlated [36]. Despite this, investigating the intake of carotenoid-rich food items is still paramount, especially for the health care system. It could offer a simple strategy for primary prevention of the disease, whereas laboratory analysis could support a further diagnosis instead. Additionally, proper calibration and adjustment for dietary habits seems plausible to reduce the risk of bias, as it was done in the latest Adventists Health Study-2 [29].
5. Studies that tested supplementation of carotenoids did not evaluate carotenoid dietary intake in any way. For example, in the CARET study, the only limitation for a participant was not to use dietary supplements containing >5500 IU/day retinol or any BC [21]. However, no evaluation of dietary habits was carried out. Therefore, a large bias due to the differences in daily intake of red and yellow vegetables (especially carrot, rich in BC) must be considered here. The same is true for the ATBC Study [23] and the Procomb trial [18].
6. Measurement of serum concentrations of carotenoids was the predominant method in the investigated studies (11 of 22). However, the course of the procedure of taking blood samples varied strongly among different studies. Only in the KIHD study was the procedure precisely described, which included: (i) definition of times for taking blood samples (between 8:00 AM and 10:00), (ii) definition of the site of sampling (antecubital vein), (iii) presampling recommendations for patients (to be after overnight fast, abstain from consuming alcohol for 3 days and from smoking for 12 days), (iv) definition of the technique for taking blood samples (blood sample was taken without tourniquet and after 30 min of resting in supine position) [15,16]. None of these aspects were discussed in nine studies [19,20,25,26,27,28,33,34] and only one aspect was mentioned in one study [17]. In different studies, the blood was taken into either heparinized tubes, EDTA-tubes, or tubes without anticoagulant. The influence of methodology of taking blood sample on measuring carotenoids was not investigated in any research. Therefore, the magnitude of potential bias from such diverging blood drawing techniques remains unknown.

**Table 1:** Studies that investigated the role of carotenoids in the prevention of prostate cancer (PC). The OR refers to the odds of developing PC when comparing the intervention group to a control group (in controlled clinical trials) or the group with minimal to the group with maximal carotenoid concentration.

Study Type	Study Name (If Given) and the First Author	Year	Nationality of Participants	PC Cases	Carotenoid	Length of the Study	Dose or Plasma Concentration (min)	Dose or Plasma Concentration (max)	Results [RR or HR, or OR (95% CI)] ^1^	Quality ^2^	Comments	Reference
Controlled clinical trial	CARET Neuhouser	2009	American ^3^	322	BC + retinyl ester	11 years	30 mg + 25,000 IU per day	N/C	0.65 (0.43, 0.97)	-	All participants were smokers	[21]
	ATBC Study Virtamo	2014	Finnish	2321	BC	18 years	20 mg per day	N/C	0.98 (0.88, 1.10)	-	All participants were smokers	[23]
Cohort study	KIHD Karppi	2009	Finnish	55	LYC	12.6 years	<4.3 μg/dL	>10.2 μg/dL	*p* >> 0.05	8	-	[16]
	ATBC Study Mondul	2011	Finnish	1732	BC Retinol	3 years	20 mg per day <48.3 μg/dL	- >68.5 μg/dL	1.19 (1.03, 1.36)	8	All participants were smokers	[25]
	KIHD Karppi	2012	Finnish	68	BC AC LYC Retinol	15 years	<13.4 μg/dL <3.2 μg/dL <4.3 μg/dL <54.1 μg/dL	>21.5 μg/dL >5.9 μg/dL >10.2 μg/dL >60.4 μg/dL	2.29 (1.12, 4.66) 2.05 (0.96, 4.36) *p* >> 0.05 *p* >> 0.05	8	-	[15]
	JACC Umesawa	2014	Japanese	143	BC AC	16 years	<0.986 mg per day <0.105 mg per day	>3.178 mg per day >0.496 mg per day	*p* >> 0.05 *p* >> 0.05	8	-	[35]
	HPFS Zu	2014	American ^3^	5728	LYC	>10 years	<36.6 μg/dL	>50.2 μg/dL	0.91 (0.84, 1.00)	8	For pre-PSA era: 0.88 (0.79–0.98)	[37]
	HPFS Graff	2016	American ^3^	884	LYC	>10 years	<3.861 mg per day	>10.262 mg per day	0.88 (0.81, 0.96)	8	For ERG (+) PC: 0.52 (0.37–0.73)	[22]
	ATBC Study Hada	2019	Finnish	2724	Retinol	>10 years	<48.3 μg/dL	>68.5 μg/dL	1.28 (1.13, 1.45)	8	All participants were smokers	[30]
	AHS-2 Fraser	2020	American and Canadian	1226	LYC	7.9 years	6.3 mg per day ^4^	5.9 mg per day ^4^	*p* >> 0.05	8	No smokers HR for canned or cooked tomatoes 0.38 (0.07–0.97)	[29]
Case-control study	PLCO Schenk	2009	American ^3^	692	Retinol	8 years	28.0–54.0 μg/dL	85.0–263.0 μg/dL	0.52 (0.32, 0.84)	9	Results only for Gleason ≥ 7	[33]
	MEC Gill	2009	multiethnic	382	BC LYC retinol β-cryptoxanthin zeaxanthin/lutein	>2 years	9.8 μg/dL 22.0 μg/dL 83.5 μg/dL 13.8 μg/dL 26.9 μg/dL	59.7 μg/dL 65.6 μg/dL 163.0 μg/dL 56.2 μg/dL 62.5 μg/dL	*p* >> 0.05 *p* >> 0.05 *p* >> 0.05 *p* >> 0.05 *p* >> 0.05	9	-	[38]
	Case-Control Surveillance Study Zhang	2009	American	1706	BC	>10 years	Only years of supplementation validated	-	*p* >> 0.05	5	Study does not allow to estimate β-carotene intake	[24]
	Beilby	2010	Australian	96	BC LYC Retinol	>1 year	5.4–16.1 μg/dL 0.0–10.2 μg/dL 31.5–82.5 μg/dL	32.8–198.6 μg/dL 16.6–69.8 μg/dL 96.0–165.9 μg/dL	*p* >> 0.05 *p* >> 0.05 *p* >> 0.05	5	All participants were smokers or exposed to blue asbestos	[17]
	PCPT Kristal	2011	multiethnic	1683	LYC	7 years	<26.3 μg/dL	>46.6 μg/dL	1.42 (1.03, 1.96)	9	Only in “not for cause” group	[26]
	ProtecT Gilbert	2012	English	1433	Retinol	4.4 years	14.3–40.1 μg/dL	63.0–117.44 μg/dL	*p* >> 0.05	7	-	[27]
	PCPT Nash	2015	multiethnic	975	BC AC retinol β-cryptoxanthin	7 years	<140.0 μg/dL <30.0 μg/dL <580.0 μg/dL <60.0 μg/dL	>370.0 μg/dL >700.0 μg/dL >770.0 μg/dL >120.0 μg/dL	*p* >> 0.05 T: *p* >> 0.05 A: 1.32 (1.01, 1.73) T: 1.30 (1.00, 1.68) A: 1.74 (1.14, 2.68) *p* >> 0.05	9	T for “total PC” A for “aggressive PC”	[34]
	Procomb Morgia	2017	Italian	9	LYC (+ Se)	2 years	5 mg per day	-	*p* >> 0.05	8	-	[18]
	Hoang	2018	Vietnamese	244	BC AC LYC β-cryptoxanthin zeaxanthin/lutein	3 years	<3920 mg per day <743 mg per day <648 mg per day <539 mg per day <1670 mg per day	>5780 mg per day >976 mg per day >1200 mg per day >867 mg per day >2580 mg per day	*p* >> 0.05 *p* >> 0.05 0.46 (0.27, 0.77) *p* >> 0.05 *p* >> 0.05	6	-	[32]
Cross-sectional study	NHANES Beyodun	2011	American	3927	BC AC LYC retinol β-cryptoxanthin zeaxanthin/lutein	N/C	0.6–7.5 μg/dL 0.2–1.4 μg/dL 0.4–12.4 μg/dL 0.7–53.0 μg/dL 0.1–4.4 μg/dL 1.7–10.8 μg/dL	21.5–343.1 μg/dL 4.8–96.6 μg/dL 27.9–80.0 μg/dL 75.1–250.9 μg/dL 12.2–150.9 μg/dL 21.1–110.9 μg/dL	*p* >> 0.05 0.38 (0.14, 1.0) *p* >> 0.05 0.49 (0.32, 0.76) *p* >> 0.05 *p* >> 0.05	-	Results refer to PSA level, not PC incidence	[28]
	Mariani	2014	Italian	9	LYC	N/C	<1 ng/mg	>1 ng/mg	not given	-	LYC concentration was measured in prostate biopsy specimen	[19]
	Nordström	2016	multiethnic	81	BC AC LYC	N/C	not given	not given	0.31 (0.15–0.63) 0.34 (0.18–0.66) *p* >> 0.05	-	-	[20]

^1^ Relative risk in all clinical controlled trials, hazard ratio in all cohort trials, and odds ratio in all case-control studies and cross-sectional studies. ^2^ According to the Newcastle–Ottawa Scale. ^3^ The mentioned nationality was most prevalent in the given study. ^4^ Estimates for participants with no PC vs. with any type of PC, respectively. Abbreviations: AC—α-carotene; AHS-2—Adventist Health Study-2; ATBC Study—The Alpha-Tocopherol, Beta-Carotene Cancer Prevention Study; BC—β-carotene, CARET—The Beta-Carotene and Retinol Efficacy Trial; HPFS—Health Professionals Follow-Up Study; JACC—The Japan Collaborative Cohort Study; KIHD Study—The Kuopio Ischaemic Heart Disease Risk Factor Study; LYC—lycopene; MEC—multiethnic cohort; NHANES—National Health and Nutrition Examination Survey; PC—prostate cancer; PCPT—Prostate Cancer Prevention Trial; PLCO—Prostate, Lung, Colorectal, and Ovarian Cancer Screening Trial; ProtecT Trial—Prostate Testing for Cancer and Treatment Trial; PSA—prostate-specific antigen.

Indeed, serum concentrations of carotenoids may differ strongly depending on the time of sampling. Among the analyzed studies, in four of them, more than one single sampling of blood was carried out [19,26,33,34]. Findings from the PCPT study suggest that a single measurement may not be sufficient for establishing a correlation to future serum carotenoid concentrations. Intraclass correlations for a given carotenoid in serum concentration over seven years were: BC 46.3%, retinol 64.2%, α-carotene 73.7%, and β-cryptoxanthin 78.7% [34]. To compare, in the ATBC study, correlation between baseline and three-year retinol measurements was 80.1% [25]. It has already been shown that the concentration of carotenoids depends, among others, on the season of year. A good example is a more than 100% higher serum retinol concentration in mobile pastoralists in Chad when sampling was taken in the rainy season, compared to the cold season [39]. This variability strongly lowers the accuracy of a single measurement for predicting later carotenoid concentration within-subjects, with the most significant impact on assessment of BC and retinol.

The least heterogeneity among studies was observed regarding the methodology of laboratory analyses. The single and only method used was high performance liquid chromatography (HPLC), as this is the routine technique for carotenoid detection and quantification, though also HPLC coupled with mass spectrometry (LC-MS) is becoming more common [40,41,42], especially for determining carotenoid metabolites. In brief, serum samples are stored at low temperature (−70 °C or −80 °C) until the moment of analysis. Then, they are extracted with hexane (sometimes ethanol, though this would not extract the more apolar carotenoids) and the sample is evaporated and then re-dissolved in mobile phase (often acetonitrile–methanol–chloroform mixtures). Next, samples are injected onto a C18 (or C30) analytical reversed phase column and detected by diode array detector. Routine wavelengths used are: for LYC 470 nm, for AC and BC 452–454 nm, for retinol 325 nm, and for β-cryptoxanthin 476 nm [15,16,20,25,26,28,34]. The quantification occurs typically via external calibration curves, employing commercially available standards, though internal standards such as trans-beta-apo-8′-carotenal are also used [40,41].

7. Studies that investigated the effects of dietary carotenoids used a slightly different way to assess their results. In the Health Professionals Follow-up Study (HPFS) study, data collection was conducted through a standardized 131-food-item semi-quantitative Food Frequency Questionnaire (FFQ). Respondents were obliged to fill out the questionnaire a total of six times to eliminate the influence of dietary habit changes during the twenty-year-long study period. Questions about frequency and type of consumed food were asked. This is an often used technique for estimating the daily intake of carotenoid-rich food items. The content of each carotenoid was then calculated by means of data from the US Department of Agriculture (USDA) food composition database [21,22], which is one of the few databases with sufficient entries regarding carotenoid content, i.e., listing various food compounds and their carotenoid profile and amount per food item. The study conducted by van Hoang et al. in Vietnam used data from the USDA database as well, but they applied only a 89-item questionnaire [32]. In the Japanese JACC study, only 35 foods were investigated. Additionally, not all of them were considered as frequently eaten in other countries (i.e., Chinese cabbage or garland chrysanthemum) [35]. This makes the results of the discussed studies less comparable, apart from the limitation of using food composition databases from other countries, as food composition is much influenced by provenience and other aspects, such as storage and processing of food items. Each of those studies were tailored toward different populations, and results should not be expanded to others.
8. The overall quality of studies was high or moderate. Controlled clinical trials [21,23] provided strictly defined intervention and control definitions as well as the endpoint. The blinding process was conducted in the proper way. The main concern is the lack of data regarding the concealment of allocation. Issues regarding assessment of the carotenoid intake or technique of diagnostic material acquisition were discussed in previous paragraphs.

All cohort studies [15,16,22,25,29,30,35,37] received 8 of 9 Newcastle–Ottawa Scale (NOS) points, as they were not able to exclude the presence of the endpoint at the beginning of the study (i.e., none of the study involved initial prostate biopsy to exclude presence of clinically silent PC). Nevertheless, the remaining NOS criteria were excellent met.

Among case-control studies, some received lower scores according to NOS scale because: the exclusion of PC in the control group was not done, the control population was selected from a specific group (i.e., patients with cardiovascular diseases), the exposure assessment was poor (i.e., the questionnaire was not standardized and validated) or they were lacking information about blinding to case/control status of interviewer, or the information about nonresponse rate was not provided. Therefore, four case-control studies [17,24,27,32] received 5–7 NOS point and their quality must be considered as moderate.

No major concerns regarding the quality of cross-sectional studies were raised—whether it must be taken into consideration, that their results could be only supportive in any sort of concluding about the relationship between carotenoids and PC incidence.

In the following, we discuss results of our findings separately for LYC, BC, and retinol. This approach was due to the different biology and absorption distribution, metabolism, and excretion (ADME) of each carotenoid. Indeed, their effects were reported to be either different or even antagonistic.

### 3.2. Carotenoids and Prostate Cancer Risk—Lycopene

Before focusing on the human studies, we will first discuss the one animal study that was dedicated to analyzing the risk of PC. It drew some conclusions that appear to be important for a better understanding of results and restrictions of the human studies. The study was conducted by Conlon et al. in 2015 and investigated whether supplementing, in a TRAMP mice model, with 10% tomato powder (TP), would influence the incidence of PC. The duration of the intervention was eight weeks. In this study, the effects of LYC and BC were examined. The only results that reached significance were inconsistent with the time trend—LYC was shown to reduce the risk of adenocarcinoma after sixteen weeks, but not in groups of mice after twelve or twenty weeks. Another finding of this research was more indicative. The tissue of distribution of carotenoids differed strongly and depended on serum carotenoid concentrations in a nonspecified way. For LYC, the liver concentration was almost linearly related to serum concentration. For gonadal adipose tissue, there was a trend found. Finally, in testes and anterior prostate (equivalent to the human prostate), LYC concentrations were constant regardless of the changes in serum concentrations. BC was not measured in tissues due to its low levels (below the limit of detection—0.5 nmol/g) [43], which is typical for mice having a much higher BCO1 activity than humans. The results questioned whether serum concentrations of LYC are a good revelator of its presence in prostatic tissue, though this was claimed in some human trials [36,44].

Among the 13 human studies investigating LYC (five cohort [15,16,22,29,37], five case-control [17,18,26,32,38], and three cross-sectional studies [19,20,28]), six presented statistically significant results [21,22,26,29,32,37]. There were three prospective studies, and two based their conclusion on the results of the HPFS, published in 2014 [21] and 2016 [22], while the remaining study was the AHS-2 [29]. In this study, the investigated cohort consisted of 48,898 male health professionals aged between 40 and 75 years at baseline. More than 90% of participants were white Americans, and a total of 5728 PC cases were followed. In a study conducted by Zu et al., LYC intake was presented according to quintiles (Q1: <3.687 mg per day; Q5: >10.131 mg per day). For the highest quintile, there was a borderline protective effect against total PC—HR, which was 0.91 (95% CI: 0.94, 1.00). The unique feature of this research was that the authors investigated differences of LYC in the pre- and post-PSA era. It was intended to check whether the effectiveness of LYC was modulated by primary prevention. Among men who underwent at least one negative PSA test at baseline, the HR for the fifth quintile was 0.88 (95% CI: 0.79, 0.98). The team concluded that high dietary LYC intake was associated mainly with a reduction in the more aggressive states of the disease, which resulted in stronger clinical symptoms. They supported the idea of additional measurements of vessel development and angiogenic marker expression in collected tumor tissue. The angiogenic score was significantly lower in the lowest quintile compared to the highest (Q1: −0.32; Q5: 0.12; *p* < 0.0007) [37]. Results of the HPFS study were also investigated by Graff et al., especially in the context of molecular subtypes of PC. The tumor with the fusion gene TMPRSS2: ERG is said to be present in about half of all PC cases worldwide and contributes to the development of PC. The authors verified whether this specific translocation influenced cancer susceptibility to LYC. The quantile ranges were very similar to the ones described above (Q1: <3.861 mg per day; Q5: >10.262 mg per day). Among the 5543 PC cases, 884 were assayed for ERG. For the last quintile, there was a similar protective effect, with an HR of 0.88 (95% CI: 0.81, 0.96). Finally, the ERG (+) PC weighted HR was 0.52 (95% CI: 0.37, 0.73) and protective effects were seen starting from the third quintile. This study proposed that LYC protected from PC more in the pre-PSA era, as HRs for each parameter in a cumulative average model were slightly higher than in the baseline analysis (HPFS was run in 1986, while using PSA as primary prevention method started in 1994). The authors suggested that a more significant reduction in ERG (+) PC incidence could explain the results of some previous studies, which showed no association for LYC. In Asian populations the prevalence of TMPRSS2: ERG translocation is less frequent, so studies that investigated Asiatic populations may not have found such a strong reverse correlation [22].

The newest report touching on the issue of tomato intake (though, indirectly of LYC) is AHS-2, covering the cohort of 27,934 men and 1226 PC cases from America and Canada, who were followed-up with over 7.9 years. The study was based upon FFQ questionnaires used to estimate daily LYC intake (with particular attendance referred to tomatoes and tomato-based products). The mean LYC intake was 6.1 mg per day in men without PC and 5.8 mg in those who developed PC of any severity (*p* = 0.174, none of the further mentioned variables were significant for this comparison). However, considering the intake of LYC derived only from canned or cooked tomatoes (0.726 mg vs. 0.625 mg of LYC, or 25.0 g vs. 21.5 g of tomatoes per day, respectively), a strong protective effect against PC was revealed (calibrated HR = 0.38 (95% CI: 0.07, 0.97), and distinctly so for the aggressive disease in the age-adjusted model (Gleason score ≥ 7; HR = 0.83 (95% CI: 0.69, 0.99), but not significantly in the multiadjusted model). The study introduced the interesting idea that the processing of LYC-containing products might in fact enhance its capability to limit the growth of PC. The authors’ suggestion was that the observed effect stems from an increased bioavailability of a heated or oil-treated LYC-rich matrix (due to the increase of its *cis*-isoforms, predominant in human blood and tissues, which may be of higher bioavailability than the all*-trans* form) [29].

Results of the HPFS study showed that dietary LYC was associated with a protection from developing PC. Results were adjusted for almost all confounding factors that could be interfering (i.e., age, nationality, physical activity, addictions, and smoking)—only alcohol intake was not included. The analyzed groups were the largest ones of those included in the present review. Also, the dietary questionnaires were taken six times, thus the changes in nutritional habits were closely monitored. The main concern was that all participants were healthcare professionals and, therefore, might not be a representative group of the whole community. Analysis for TMPRSS2: ERG associations was naturally limited only to subjects who underwent a radical prostatectomy, as this specific translocation is acquired in prostatic tissue, while dysplastic changes are further developing. It is worth noting that a reverse association with PC was even stronger for tomato sauce than for LYC alone, as shown by Graff et al. [22]. As the LYC intake itself was calculated only based on FFQ results, it could be theoretically possible to overestimate the influence of LYC, assuming there are other protective agents in food, such as dietary fiber, polyphenols, vitamin E, or other. On the other hand, it is possible that LYC absorption and metabolism is merely supported by other active compounds present in vegetables and vegetable-based food items. One needs to consider also the food processing, which, according to the results of the AHS-2 study, are a crucial factor determining results of dietary LYC [29]. Data obtained from such studies may be of particularly practical potential, as they could be translated more directly into dietary recommendations.

Out of the three retrospective studies, one found a positive correlation between LYC and PC, however it still referred only to one specific type of PC—detected despite any indication by biopsy (the PSA level and DRE were normal). It was based on results from the Prostate Cancer Prevention Trial (PCPT), which originally tested the efficacy of 5 mg per day of finasteride, an 5-α-reductase type 2 inhibitor, for PC prevention. The study assessed LYC serum concentrations but also included prostate biopsies collected every year, which enabled researchers to avoid bias due to the PSA-dependent underdiagnosis of the disease. Almost 90% of cases were white Americans, thus the ethnic structure was similar to that of the HPFS study. LYC concentrations were stratified by quartiles (Q1: <26.3 µg/dL; Q4: >46.6 µg/dL). In the placebo arm, the only statistically significant result was a higher risk of PC not found by PSA screening in the fourth quartile—HR was 1.42 (95% CI:1.03, 1.96). Interestingly, in the finasteride arm, the frequency of PC detected due to the raised PSA was lowered; HR was 0.57 (95% CI: 0.34, 0.95). Simultaneously, if already diagnosed for cause, the disease was more aggressive and presented a higher grade or stage. There were about 50% more cases of PC in Gleason stages 7–10 in the for cause group than in the not for cause group [26].

In a somewhat disturbing conclusion, it was thought that the protective effect of LYC might merely be due to lowering the sensitivity of the PC screening instead. Not-for-cause-detected tumors in PCPT comprised 46.8% of the total cases. In the highest quartiles of LYC, the quotient of risks of not for cause/for cause were increasing strongly. It cannot be compared to any other study, as prostate biopsies have not been used commonly in studies. It is possible that LYC could, in fact, impair detection of a low-grade disease, which would not turn out to be harmful even in long-term perspective.

Another case-control study was carried out in Vietnam by van Hoang et al. in 2013-2015 [32]. Within this study, data about dietary habits of citizens of Ho Chi Minh city were collected, using the information to calculate average LYC intake. The FFQ used in this study was comprised of 89 food items—it is worth underlining that tomato sauce (found as the most effective source of LYC in the aforementioned studies) was excluded from that questionnaire as it is not popular in the Vietnamese population. The population studied comprised men aged 64–75 years. Among the 652 participants, 244 PC cases were described. Results were presented as tertiles (Q1: <0.648 mg per day; Q3: >1.2 mg per day). It is an interesting observation that, despite the fact that the highest tertile had a three-times lower risk than the lowest in HPFS, the protective effect of LYC remained significant and strong here. The value of OR for the third tertile was 0.46 (95% CI: 0.27, 0.77). For tomatoes, there was an observed 61% reduction of PC risk in the third tertile. It also turned out that all protective effects contributed to the low grade disease, as for PC with a Gleason score ≤ 7, OR was 0.41 (95% CI: 0.21, 0.77) and no correlation was shown for the aggressive disease.

Despite the results being generally in agreement with the findings of the HPFS, there are some differences that impede the comparison of their conclusions. First, the Vietnamese study was retrospective and, in addition, data were extracted from the population of a single city. There were also significant changes between the questionnaires (e.g., exclusion of tomato sauce) compared to the HPSF. Interestingly, this study put into a question the assumption that in Asiatic populations the effect of tomatoes and diets high-in-lycopene is weaker than in American or European populations. Given that the intake of carotenoids in this study was much lower than in American studies, it may even suggest some U-shaped curve of dependency. The narrow range of the study allows us only to state that LYC might protect from low-grade PC in the Vietnamese population.

The last study of LYC was the North Carolina–Louisiana Prostate Cancer Project (PCaP), a cross-sectional study investigating, separately, the role of LYC in European Americans (EA) and African Americans (AA), aged 40–79 years. Dietary habits were collected based on the National Cancer Institute’s Diet History Food-Frequency questionnaire (NCI-DHQ), containing 144 common food items. Additionally, data about carotenoid supplement intake were taken. Results were presented as tertiles (Q1: <3.605 mg per day; Q3: >6.299 mg per day). In this study, there was a protective effect of LYC against aggressive PC (defined as Gleason ≥ 8) and the OR for the third tertile was 0.55 (0.34, 0.89). However, it referred only to the EA group; as for the AA, they found no significant associations [45]. Due to the cross-sectional character of the study, we could only conclude that EA people with diagnosed high-grade PC had a lower dietary intake of LYC at the time of cancer diagnosis.

When summarizing our conclusion for LYC it can be stipulated that:

1. Increased consumption of LYC from canned or cooked tomatoes might be related to a reduced hazard of developing PC (e.g., 25 g vs. 20 g per day of tomatoes reduces hazard by ~60%) and, supposing, aggressive PC;
2. Intake of more than 10 mg of LYC was related to a reduced risk of diagnosing PC, by at least 10%, compared to an intake below 3.6 mg per day in European and American populations;
3. For men with TMPRSS2: ERG translocation, the reduction in risk of diagnosing PC reached about 50%;
4. LYC was associated with an increased risk of developing PC without abnormal PSA or DRE by about 40%—however, this subgroup of PC is actually undetectable (by definition) due to the lack of clinical signs;
5. The clinical significance of underdiagnosed PC cases must be evaluated—if they remain unharmful over time, it would be beneficial from a public health point of view not to detect their presence—reducing the postoperational burden of patients and costs of healthcare;
6. In the Vietnamese population, an intake of 1.2 mg of LYC per day was associated with a reduced risk of diagnosing PC by about 50% compared to a daily intake of less than 0.648 mg.

### 3.3. Carotenoids and Prostate Cancer Risk—BC

There have been twelve studies that focused on BC. However, their comparability is low. Two were cross-sectional [20,28], five were case-control [17,24,32,34,38], three were cohort studies [15,35,38], and two were intervention trials [21,23]. Firstly, we will discuss the Carotene and Retinol Efficacy Trial (CARET) and the Alpha Tocopherol, Beta Carotene Cancer Prevention Study (ATBC) as they were both rather clinical-controlled intervention trials. In CARET, participants were supplemented with 30 mg of BC and 25,000 IU of retinyl palmitate every day for an average of 11 years. In this study, a total of 890 PC cases were reported. It must be highlighted that all participants of the study were smokers or exposed to asbestos in the past. There was a subgroup of participants who additionally took commercial dietary supplements. The results were presented separately for the intervention and postinterventional phase (follow-up) of the study. Significance was reached only for the intervention part. For the active study arm without supplements, RR for nonaggressive (but not total) PC was 0.65 (95% CI: 0.43, 0.97), compared to placebo without supplements. In the active arm of the study there was a slightly higher prevalence of high-grade PC (Gleason score ≥ 7) in comparison to the placebo group (44.6% versus 40.1%) [33]. In the ATBC study, Finnish men, aged 50–69, received 20 mg of BC daily for an average of six years (the group that was supplied only with tocopherol will not be discussed here). In total, 2321 cases of PC were evaluated. Again, in this study all subjects were smokers. This study did not show any significant correlation between BC and PC as the RR was 1.03 (95% CI: 0.95, 1.12). The post-trial analysis also showed no associations [23]. Both of these prospective studies referred only to excessive smokers (people who smoked around 20 cigarettes a day). On the one hand, the protective effects shown in CARET may be due to the higher dosage of BC than those used in the ATBC study. On the other hand, the effects of retinyl esters should likewise not be underestimated. As the protective effect disappeared in the follow-up, it may be proposed that only continuing extensive supplementation with BC and retinyl palmitate lowered the risk of PC.

Another large-scale study also investigating the Finnish population was The Kuopio Ischaemic Heart Disease Risk Factor Study (KIHD). This trial evaluated serum levels of BC in patients aged 42–60 years at baseline. The second measure was taken at the end of the study. Mean concentrations were divided into tertiles (Q1: <13.4 µg/dL; Q3: >21.5 µg/dL). This was the only study that provided full data on the blood sampling procedure, which allowed us to largely exclude diagnostic bias in this study. A total of 68 PC cases were registered. In this study, cases were significantly older than those without PC—means for age were 58.9 and 55.9, respectively (*p* < 0.001). Among these cases, significantly higher concentrations of α-linoleic (ALA) were observed, which was meant to be a covariate for results (elevated in PC cases). The difference in plasma BC levels was also significant, although less remarkably. Mean concentrations were 24.7 and 20.4 µg/dL, respectively (*p* < 0.04), however BC concentration varied, depending on the month of sampling, with highest concentrations between July and September. This study observed a great increase in PC risk for higher BC concentrations. The RR for the third tertile in comparison to the first was 2.29 (95% CI: 1.12, 4.66) in a fully adjusted model (adjustment covered age, examination year, family history of cancer (yes vs. no), BMI, years of smoking, alcohol consumption, education, physical activity, serum total cholesterol, and serum ALA) [15]. This nested case-control study should be carefully considered. The small sample size, a few factors that differed between cases, and unaffected participants suggests some risk of bias. In addition, the whole group was from one city, and thus they might not be representative for the general Finnish community. Finally, seasonal and personal fluctuations in BC levels seemed to be a possible confounder in such a small trial.

The fourth study was conducted by Nordström et al. and analyzed circulating carotenoids, PC risk, and their relation to the genomic instability in a population of 81 Caucasian men. The concentrations were given in quartiles. In total, 20 of the SNPs in SOD1-3, XRCC1 and OGG1 genes were measured. In this study, men with high-grade tumor (Gleason score ≥ 7) were preferably chosen. Comparing the highest versus the lowest quartile, OR for diagnosis of a high grade of PC was 0.31 (95% CI: 0.15, 0.63) in the fully adjusted model (adjustment for age at diagnosis (years) and circulating cholesterol (mg/dL), smoking status at diagnosis (ever vs. never), and Caucasian origin). It is worth noting that nonadjustment for smoking resulted in both higher OR value and a lower *p*-value—suggesting again an inverse association between BC and cigarettes. Interaction with SNPs turned out to be beneficial for the TC/CC genotype of rs699473 in the SOD3 gene. The OR value for the risk of high grade PC in Q4 versus Q1 among TC/CC genotype carriers was 0.20 (95% CI: 0.09–0.45) [20]. This study undoubtedly introduced the idea of strongly differentiated molecular susceptibility to carotenoids, especially to BC. This may raise caution for highlighting the results of this study due to the lack of presenting quartile ranges used in analysis.

In summary, none of the prospective studies met the requirements to be used as a source of any strict conclusions (e.g., due to the subjects being nonrepresentative for the whole community). Simultaneously, the study by Nordström et al. comprised not enough cases and its retrospective character could be a source of bias. Based on the collected data, we can only derive certain partial conclusions:

1. In smokers, constant supplementing 30 mg of BC (together with 25,000 IU retinyl palmitate) each day may decrease the risk of developing nonaggressive PC;

2. Maintaining a higher serum BC level might decrease the risk of high grade PC, particularly among people with the TC/CC variant (rs699473) in the SOD3 gene. On one hand, SOD3 plays a significant role in ROS handling. On the other hand, free radicals react easily with BC. It could provide an explanation for the correlation of SOD3 polymorphisms to protective effects of BC.

### 3.4. Carotenoids and Prostate Cancer Risk—Retinol and Retinyl Esters

Nine studies included in our review evaluated the effectiveness of retinol toward the prevention of PC. These included one cross-sectional [28], five case-control [27,28,30,33,34], and three cohort studies [15,25,30]. Four of them reached a statistically significant outcome. The placebo-controlled trial, CARET, which investigated effects of long-term supplementation of retinyl palmitate together with BC, was described and discussed in the previous chapter.

Three case-control studies referring to serum concentration of retinol were nested in the Prostate, Lung, Colorectal, and Ovarian Cancer Screening Trial (PLCO) within the ATBC study and in the PCPT. The PLCO involved 692 cases of PC (cases of non-Hispanic Black men were excluded from analysis). Concentrations were given in quintiles (Q1: 27.4–54.7 μg/dL; Q5: 85.4–262.6 μg/dL). Statistically significant results were found for risk of high-grade disease (Gleason score ≥ 7) in Q1 versus Q5, with an OR of 0.52 (0.32, 0.84). For each quintile, a protective effect was shown (compared to the first one). A correlation with clinically aggressive disease stage (III or IV) could not be demonstrated. What weakens the impact of the results from this study is that retinol concentration was measured only once, at the baseline of the study. For a small subgroup of 46 men, assessment was repeated one year after baseline and only a moderate correlation with initial retinol concentration was found (r = 0.38) [33]. The ATBC study design has been described in the previous section. In this nested case-control study by Mondul et al., baseline and three-year serum retinol were measured. In total, 1732 cases of PC in Finnish smokers supplemented with a daily dose of 20 mg of BC were collected. During the three-year course, correlation of retinol concentrations were 80.1% between the baseline and three-year measurement. The results were given in quintiles (Q1: <48.3 μg/dL; Q5: >68.5 μg/dL). The highest concentration of retinol was associated with a higher risk of total PC, both for the baseline and three-year follow-up. HRs were 1.19 (95% CI: 1.03, 1.36) and 1.22 (95% CI: 1.05, 1.41), respectively. The authors of the study emphasized that although all participants of the ATBC study were smokers, adjusting for pack-years, the duration of smoking or number of cigarettes smoked every day did not change this outcome significantly [25]. These results were also obtained following a reanalysis by Hada et al. in 2020, who concluded that HRs were actually higher after adjustment for multiple variables (HR = 1.28 (95% CI: 1.13, 1.45) *p* < 0.0001). In fact, exclusion of participants diagnosed within two years of blood sample collection (i.e., reducing the risk of bias due to the diagnosis of already developing PC at the initial point of the study) slightly increased HR [30].

The PCPT design was also described in the previous chapter. In this investigation by Nash et al., a positive association of serum retinol and aggressive PC was shown. At the end of the study, all participants underwent prostate biopsy, resulting in 54.1% of total PC cases. Measurements of retinol concentrations were done annually. Average results were given as quartiles (Q1: <58 μg/dL; Q4: >77 μg/dL). An increased risk of total PCa, as well as aggressive disease (Gleason score ≥ 7), was shown—ORs (Q1 versus Q4) were 1.43 (95% CI: 1.02, 2.01) and 1.91 (95% CI: 1.10, 3.31) respectively. Moreover, results were stratified depending on whether PC was detected for cause (indicated by DRE or PSA) or not for cause. The highest serum retinol levels resulted in a 145% increased incidence of aggressive PCa (95% CI was 1.24, 4.85). All these associations were seen only in the placebo arm of the PCPT (men who had not been receiving finasteride) [34]. As it was previously discussed, in the PCPT, it was shown that LYC increased the risk of clinically undetectable PC, particularly the nonaggressive disease. For retinol, somehow contrasting results were found—an increased risk of high-grade PC diagnosed for cause. This led to the hypothesis that different carotenoids may not so much affect the risk of PC itself but rather modifying the chance to detect the tumor, through interfering with, for example, concentrations of PSA. The NHANES study investigated the influence of retinol on PSA level, however it did not reach any significant result in a fully adjusted model [28].

It is of high importance to mention the discoveries of novel research conducted with participants of the PLCO study (4662 cases and 3114 controls—substantially more than in previously published results from this trial). From 31 investigated genes involved in retinol metabolism (most of which were discussed in the part I of this review [1]) SNPs of two genes were significantly more frequent in patients with PC, namely rs1330286 of ALDH1A1 (OR = 0.88, 95% CI: 0.83, 0.94; the expression quantitative trait loci (eQTL) of this gene) and rs4646653 of ALDH1A3 (OR = 1.17, 95% CI: 1.07, 1.27), with increased ALDH1A3 mRNA in PC tissue specimens than in healthy controls [46]. For years considered as the marker of PC stem cells, now ALDH1A1 gains interest as the link between retinol and PC. This is supported by results of laboratory investigations—e.g., its inhibition by sylibin resulted in reduction of RARα in DU-145 cells and prevention of proliferation, migration, and invasion [47]. The frequency of ALDH1A1 polymorphism was equal to 45% in PC group.

Thus, there are not sufficient data to develop a strict conclusion on the effectiveness of retinol regarding the prevention of PC. Referring to the results of the PLCO and the ATBC trials it could be stated that:

1. In smoking subjects with high retinol serum concentrations, there is a 20–30% increased risk of total PC;

2. Retinol might increase the risk of detecting high-grade PC, but it is uncertain whether it increases the growth of tumor (unfavorable effect), whether it merely causes increases in PSA concentrations, or enables a more sufficient primary prevention (beneficial effect).

### 3.5. Carotenoids and PC Risk—General Conclusions

The relationship between carotenoids and PC appears heterogeneous and complex. Results of the largest, prospective trials suggest that dietary LYC is protective against PC. For other carotenoids, such as BC and retinol, the relation becomes less obvious, though there is accumulating evidence suggesting that retinol, at least in subjects with distinct ALDH1A1/3 SNPs, exerts adverse effect.

Molecular and genetic differences may result in varying response to carotenoids. Lifestyle and ethnicity seem to strongly modify it as well. Finally, the significance and diagnosis of PC depends strongly on primary prevention methods. Therefore, the vast majority of discussed studies were, in fact, more directly related to PSA than to PC itself. Discussions for AC, zeaxanthin/lutein, and β-cryptoxanthin will not be carried out in the present review as there are insufficient data on their effectiveness in reducing the risk of PC to perform any comprehensive analysis.

## 4. Treatment and Survival in PC and Carotenoids

Apart from their influence on cancer risk prevention, carotenoids are vastly researched in the area of cancer treatment, after the development of disease. The aim of this section was to discuss the effects of carotenoid intake on PC treatment outcomes. In our search for trials referring to this question, we identified 19 papers that fitted the inclusion criteria as described in the method section. Among those, there were four clinically controlled trials, three cohort studies, one cross-sectional study, and five case-series reports. Two studies were nested in large randomized controlled studies (RCTs) and one was nested in a cohort study. Apart from this, we identified six experimental animal trials that evaluated the influence of carotenoids on PC tumor growth. Due to the lack of consistent methodology and endpoints, we did not perform a meta-analysis of the gathered papers (Table 2).

There have been no consistent results favoring positive effects of carotenoids in PC treatment. Moreover, it must be considered that the existence of statistically significant outcomes in studies may not reflect the real association, but also bias, resulting from performing additional analysis in the pursuit of positive results.

Furthermore, only one of the clinically controlled trials, carried out by Margalit et al. [48], was of high quality—with good blinding, well-defined intervention, hard endpoint of prostate-cancer-specific death (PCSD) and long follow up. Others in turn based their analysis mainly on PSA changes after administration of a tomato-rich product, with no blinding at all [49,50].

**Table 2:** The summary of findings of studies which investigated the administration of carotenoids in the management of PC.

Study Type	Study Name (If Given) and the First Author	Year	Nationality of Participants	PC Cases	Carotenoid	Length of the Study	Dose or Concentration	Evaluated Endpoint	Result[HR(CI) Where Applicable or in accordance to the Endpoint Type]	Quality ^1^	Comments	Reference
Controlled clinical trial	[ECOG 3899] DiPaola	2010	multiethnic	70	13cRA + IFNα2b + paclitaxel vs. vinorelbine + mitoxantrone + estramustine	8 weeks	1 mg/kg twice a week	PSA response rate disease response rate (CR + PR) median overall survival	23% (11%, 38%) vs. 50% (34%, 66%) 15% vs. 14% 13.9 mo. vs 9.4 mo.	2	Efficacy of two combination therapy assessment.	[51]
	PHS Margalit	2012	American	383	BC	10.5 years follow-up	50 mg on alternate days while receiving radiotherapy	PCSD	*p* < 0.05	8	Study nested within the PHS RCT All patients were diagnosed with PC and undergoing RT	[48]
	Paur	2014	Norwegian	79	LYC (tomato products)	3 weeks	30 mg per day	PSA change (postintervention) PSA vs. control	0.00 (−3.30, 2.40) ^2^ −0.23 (−1.12, 1.90) ^3^ 0.45 (−3.30, 4.80) ^4^ −0.02 (−2.40, 1.70) ^5^ *p* = 0.016 ^4^ *p* = 0.009 ^5^	3	All patients were diagnosed with nonmetastatic PC scheduled for radical prostatectomy or high-dose radiotherapy.	[49]
	Graingner	2019	not specified	55	LYC (tomato soy juice)	3–5 weeks	~41.2 mg per day	PSA slope differences	*p* >0.05	2	All men were diagnosed with PC and scheduled for prostatectomy. All participants received 0, 1, or 2 cans of juice for 24 (± 4.6) days prior to the surgery.	[50]
Cohort study	ATBC Watters	2009	Finnish	1891	BC (supplementation for 5–8 years) BC(baseline serum concentration) retinol(baseline serum concentration)	3 years (follow-up)	20 mg per day <105 mg/L 105–155 mg/L 156–210 mg/L 210–299 mg/L >299 mg/L <493 mg/dL 493–554 mg/dL 555–613 mg/dL 614–691 mg/dL >691	PCSD	1.02 (0.56, 1.84) ^6^ 0.96 (0.58, 1.57) ^7^ 1.17 (0.57, 2.39) ^8^ 1 (ref) 1.07 (0.78, 1.46) 1.04 (0.76, 1.43) 0.80 (0.58, 1.12) 1.01 (0.74, 1.39) p_trend_ = 0.49 1 (ref) 1.13 (0.82, 1.56) 1.20 (0.88, 1.64) 1.10 (0.79, 1.52) 1.17 (0.84, 1.63) p_trend_ = 0.44	8	Research nested in ATBC RCT. All participants were smokers.	[52]
	Venkitaraman	2010	English	143	BC AC LYC retinol (plasma concentrations evaluated)	2.5 years (median follow-up)	N/C	Time of disease progression Correlation with baseline PSA Correlation with PSA velocity Adverse histology on repeat biopsy	*p* >> 0.05 for all measures	6	Only PC patients characterized by stage T1/2a N0/Nx, PSA levels <15 ng/mL, composite Gleason score ≤ 7, primary grade ≤ 3, and percentage of positive biopsy cores ≤ 50% of total cores and not undergoing current therapy.	[53]
	CPS-II Nutrition Cohort Study Wang	2016	American	5018	LYC (estimated daily consumption)	10.2 years (mean)	<0.3 mg per day ^9^ 0.3–40.0 per day ^9^ 4.2–6.1 per day ^9^ 6.1–30.2 per day ^9^ <3.1 mg per day ^10^ 3.1–4.4 per day ^10^ 4.4–6.2 per day ^10^ 6.2–40.9 per day ^10^ low/low ^11^ low/high ^11^ high/low ^11^ high/high ^11^	PCSD	p_trend_ = 0.92 ^12^ p_trend_ = 0.92 ^13^ p_trend_ = 0.59 ^14^ p_trend_ = 0.23 ^12^ p_trend_ = 0.21 ^13^ p_trend_ = 0.50 ^14^ 1.00 (ref) ^14^ 0.80 (0.31, 2.06) ^14^ 0.72 (0.28, 1.87) ^14^ 0.41 (0.17, 0.99) ^14^		Research nested in the CPS-II Nutrition Cohort study.	[54]
Cross-sectional	PCaP Antwi	2016	American	2102	BC LYC α- cryptoxanthin β-cryptoxanthin zeaxanthin and lutein (estimated intake) BC AC LYC ATRA zeaxanthin α- cryptoxanthin β-cryptoxanthin (adipose tissue level)	N/C	N/C	Correlation of intake with PC aggressiveness ^15^ Correlation of adipose tissue carotenoid level and PC aggressiveness ^15^	*p* < 0.05 only for β-cryptoxanthin in African Americans and LYC in European Americans *p* > 0.05 for all measures	6	Research subjects subdivided into African Americans and European Americans for analysis	[45]
Case series	Cheung	2009	American	23	fenretinide	1 year	900 mg/m^2^ twice daily for one week every three weeks	PSA decline >50% PSA-stable disease Time to PSA progression Probability of having no PSA progression in 6 mo.	0% 30% 4.6 mo. (median) 0.37 ± 0.10	-	Classified by authors as modest clinical activity Patients with confirmed rising PSA ≥ 2 ng/mL, following radical prostatectomy and/or pelvic radiation therapy, without evidence of metastasis	[55]
	Schwenke	2009	German	17	LYC	6 months	14 mg per day	PSA decline >50% PSA-stable disease	0% 29%	-	PC patients treated with hormone ablation with progressive HRPC defined as increase in 3 x (+) PSA or clinically verifiable prostate cancer progression	[56]
	Moore	2012	Australian and Asian	27	fenretinide	median number of cycles—2	900 mg/m^2^ twice daily for one week every three weeks	PSA decline >50% PSA-stable for 6 weeks Time to treatment failure	4% (max. 39 days) 52% 54 days	-	Patients characterized with castrate levels of testosterone and a rising PSA concentration greater than 10 ng/mL	[57]
	Pili	2012	American	4	13cRA + etinostat	various period of intervention	1 mg/kg twice daily for three weeks every four weeks	Clinical response	In one patient with CRPC (lasted 15 mo.)		Research conducted among 19 patients with various cancers.	[58]
	Di Bella	2013	not given	16	retinoids + chemotherapy ^16^	review of period >5 years	ATRA (46 kIU) retinol (25 kIU) BC (93 kIU) [αTC (38 IU)] ratio 1:1:4:2	Percentage of patients with overall response Percentage of patients with complete response Percentage of patients with objective clinical benefit	69% (44%, 86%) 88% (41%, 93%) 50% (22%, 79%) 44% (23%, 67%) 83% (22%, 79%) 25% (7%, 59%) 88% (57%, 93%) 100% (CI N/C) 75% (CI N/C)	-	-	[59]
Animal in vivo trial	Lindshield	2010	Copenhagen rats	119	LYC	4–6 weeks before tumor implantation and ~18 weeks after that	250 mg/kg per day	Final tumor area, tumor weight, tumor weight/body weight ratio	no change (*p* not given)	-	Rats implanted with androgen-sensitive Dunning R-3327H prostatic adenocarcinoma.	[60]
	Yang	2011	Athymic nude mice	24	BC LYC	7 weeks	16 mg/kg per week 4 mg/kg per week	Tumor volume reduction Tumor mass reduction	*p* < 0.001 for all measures (60–70% reduction)	-	Mice implanted with androgen-insensitive PC-3 prostate cancer cell	[61]
	Tang	2011	Nude mice	32	LYC LYC + docetaxel	~40 days	15 mg/kg per day	Growth rate of DU145 tumor vs. control vs. docetaxel Enhancement of docetaxel antitumor efficacy	*p* < 0.05 for all measures (reduction) ↑38% *p* = 0.042	-	Mice implanted with androgen-insensitive DU145 prostate cancer cells	[62]
	Du	2016	Nude mice	56	LYC torulene torularodin	2 weeks before implantation to the end of the study	9 or 18 mg/kg per day (each)	Tumor growth inhibition	Significant changes reported for all measures, but no quantitative data given	-	Mice implanted with androgen-insensitive PC-3 prostate cancer cells	[63]
	Ni	2017	Nude mice	40	astaxanthin	2 weeks	25 mg/kg 100 mg/kg per day	Tumor volume reduction	no change *p* < 0.01	-	Mice implanted with androgen-insensitive PC-3 prostate cancer cells	[64]
	Rowles	2020	TRAMP mice	56	LYC	1 week prior to and/or ~2 weeks after castration ~2 weeks after castration	10% powdered LYC for 0.47 g 10% lycopene/kg for	Difference in tumor volume between groups	*p* > 0.05 *p* >> 0.05	-	Mice that developed PC were eligible for analysis (70% and 66% in study 1 and 2). Additionally PC incidence in animals receiving LYC and control diets was compared (*p* > 0.05)	[65]

^1^ According to the Newcastle–Ottawa Scale. ^2^ Among interventional group. ^3^ Among postsurgery patients classified as intermediate risk in the interventional group. ^4^ Among interventional group patients with low (<median) increase in serum lycopene concentration (based on median change in plasma/ped blood cell concentrations). ^5^ Among interventional group patients with high (>median) increase in serum lycopene concentration (based on median change in plasma/ped blood cell concentrations. ^6^ During trial. ^7^ 6-years post-trial period. ^8^ 12-years post-trial period. ^9^ Assessed prediagnostic daily intake. ^10^ Assessed postdiagnostic daily intake. ^11^ Prediagnostic/postdiagnostic assessed daily lycopene intake, low<median<high. ^12^ For patients diagnosed with T1–T2 PC with unknown Gleason score not included in lower risk or high-risk categories. ^13^ For patients diagnosed with T1–T2 or Gleason score 2–7 PC. ^14^ For patients diagnosed with PC characterized as T3–T4 op Gleason score 8–10, or with nodal involvement. ^15^ Defined as Gleason sum ≥8 or PSA >20 ng/mL or Gleason sum ≥ 7 and clinical stage T3–T4 PC. ^16^ Treatment protocol consisting of: somatostatin, melatonin, retinoids solubilized in α-tocopheryl acetate, D2R dopamine agonists, androgen inhibitors, and cyclophosphamide. Abbreviations: 13cRA—13-cis-retinoic acid; AC—α-carotene; ATBC—Alpha-Tocopherol, Beta-Carotene Cancer Prevention; ATRA—all-trans-retinoic acid; CPS-II—Cancer Prevention Study II; BC—β-carotene; C/HRPC—castration/hormone-resistant prostate cancer; ECOG 3899—Eastern Cooperative Oncology Group 3899; HR—hazard ratio; IFNα2b—Interferon α-2b; LYC—lycopene; PCaP—Prostate Cancer Project; PHS—Physicians’ Health Study; PC—prostate cancer; PCSD—prostate-cancer-specific deaths; PSA—prostate-specific antigen; TC—tocopherol; TRAMP—transgenic adenocarcinoma of the mouse prostate.

Regarding cohort studies, the one conducted by Watters et al. [52] seems to have been well designed, with hard endpoints (PCSD), a large cohort, and well characterized differences between participants in terms of evaluated carotenoid (BC) intake. On the other hand Wang et al. [54] estimated LYC consumption in participants based on a self-reported questionnaire, which may be a source of bias. At last, Venkitaraman et al. [53] evaluated the association between PC course and carotenoid plasma concentration, which does not necessarily reflect the actual intake of these microconstituents. The only identified cross-sectional study, carried out by Antwi et al. [45], again relied on questionnaire-based carotenoid intake and tissue levels.

As other papers were either case series or animal studies (Table 2.), there clearly is a scarcity of high-quality trials evaluating the influence of carotenoids in PC.

In an RCT by Paur et al. [49], the effect of supplementing tomato-based products standardized for LYC content on PSA change in PC patients was evaluated. Although no effect was seen among prerandomized groups, in post-hoc analysis, a decrease in PSA level was noted in PC patients postsurgically classified as an intermediate-risk group. This would suggest that the outcome of an LYC intervention depends on cancer characteristics, in line with in vitro experiments, as discussed in part I of the review [1]. Paur et al. [49] also showed that PSA change was independently most prominent in patients with the highest increase in LYC serum concentrations. Lack of consistency between no effect seen in an entire intervention group and a positive outcome in subjects with the highest increases in LYC serum concentration may be caused by the. population variability of LYC metabolism, which is supported by research demonstrating differences in carotenoid metabolic pathways in relation to their final biological effect (SOD, BCO etc.). Alternatively, a higher increase in serum LYC may be associated with increased consumption of LYC-rich food, which typically contains other biologically active substances that may act independently, confounding results. It is important to realize that questionnaire-based trials aim to assess the consumption of carotenoids and, in this study, in the subjects consuming the same tomato-based product but also consuming black/green tee, pomegranate and grape juice, soy isoflavones, selenomethionine, and n-3 fatty acids, a weaker effect on PSA was observed.

In another interventional trial, Grainger et al. found no significant difference in PSA slopes between PC patients receiving tomato-soy juice prior to the prostatectomy and a control group [66]. Wang et al. [54] evaluated the effect of LYC intake on PCSD. Although no association was found in preliminary analyses, they found fewer PCSD in a group with high LYC intake, both based on pre- and postdiagnostic questionnaires. One possible explanation is that such patients followed healthier lifestyle habits, including diet, which again emphasizes the problem of confounding factors in questionnaire-based studies. Furthermore, studies carried out by Watters et al. [52] and Margalit et al. [48] did not indicate BC or retinol supplementation and serum concentrations to be associated with PCSD, although both interventions lasted approximately from three up to ten years. Antwi et al. [45] performed a vast analysis of different carotenoids, estimating daily intake and adipose tissue concentrations and their link to the aggressiveness of disease in PC patients. Research subjects were divided into two groups: African Americans (AAs) and European Americans (EAs). Interestingly, the significant inverse correlation between PC aggressiveness and β–cryptoxanthin intake was found only in AAs, whereas in EAs, such an association was present only for LYC intake as well as LYC and AC adipose tissue concentration. Although these results are again subject to confounding factors, they also raise the possibility of an ethnicity specificity of the influence of carotenoids on health. Conversely, a cohort study conducted by Venkitaraman et al. [53] found no association between LYC, AC, BC, and retinol serum levels and time of progression, baseline PSA level, and PSA velocity in English PC patients.

Apart from assessing their role as intervention agents in cancer treatment alone, some attention has been drawn to the potential of employing carotenoids in combination therapy. DiPaola et al. [51] conducted an RCT to evaluate the relative efficacy of two multidrug regimens in the treatment of PC, one of which included 13-*cis*-retinoid acid (13cRA). However, as their work did not focus on retinoids themselves, no strong conclusion may be drawn from their study for this review. Similarly, Di Bella et al. [59] utilized an authorial treatment protocol including ATRA in PC management, and Pili et al. [58] reported on PC patients treated with a combination of entinostat and *cis*-retinoic acid.

More conclusive results may be drawn from experimental animal testing. Among six of the reviewed studies, two carried out by Lindshield et al. [60] and Rowles et al. [65] did not report favorable results of carotenoid (specifically LYC) treatment. In pursuit of the cause of this ambiguity, it is noteworthy to mention that the first study was conducted on Copenhagen rats implanted with androgen-sensitive Dunning R-3327H prostatic adenocarcinoma, while other studies (with positive results) utilized nude mice injected with androgen-insensitive tumor (PC-3 or DU145) cells. On the other hand, Rowles et al., based their results on TRAMP mice experiments that spontaneously developed castration-resistant prostate cancer (CRPC) during the LYC dietary intervention [65]. Importantly, it was shown that animal models differ in carotenoid metabolism, which may influence the results [67], notably the much higher BCO1 activity in mice. Alternatively, differences in androgen sensitivity may alter carotenoid treatment outcomes. However, studies in humans, enrolling either patients with hormone-refractory or hormone-sensitive PC, have not supported this potential difference. Four remaining animal trials reported tumor growth inhibition induced by LYC, BC, torulene, torularhodin, and astaxanthin [61,62,64,68]. Additionally, Tang et al. [61] presented an 38% increase in docetaxel efficacy against PC upon addition of all-*trans* LYC to the regimen. The unambiguity seen between trials in animals and humans may originate from uncontrolled environmental influence in the latter case. This is consistent with an analysis performed by Druesne-Pecollo et al. [69], who further suggested that the dosage of carotenoids plays an important role, with the higher dose even being positively correlated with cancer development. For instance, carotenoids such as canthaxanthin and astaxanthin, as well as β-apo-8′-carotenal, were shown to induce xenobiotic metabolizing CYP1A1 and CYP1A2 enzymes in rat livers, whereas BC and LYC had no effect [70]. On the other hand, plasma carotenoid levels were correlated with CYP1A2 activity—positively in the case of LYC and negatively in the case of lutein [70]. Those two biological effects may point out to a complex interaction between carotenoids and environmental exposure associated with the final outcome of carotenoids and human health and disease modulation, similar to that proposed by the CARET and ABTC studies [71].

In conclusion, current evidence does not allow drawing conclusions regarding the relation of carotenoids and PC prognosis. However, the aforementioned research highlights the need for careful evaluation of differences in action of distinct carotenoids in distinct populations and even among different patients. Furthermore, unambiguity between studies exist, especially between humans and animal models, emphasizing the importance of confounding factors.

## 5. Carotenoids and Prostatic Physiology and Pathology (Other Than PC) in Human Studies

All the human studies identified in regard of this topic referred to the LYC. Commonly consumed tomato products, such as tomato soup, sauce, and juice in daily servings, can increase and prostate LYC concentrations in men with PC after three weeks of a diet rich in tomato products [53]. While the majority of dietary LYC is typically in the all*-trans* geometric configuration, tissue LYC is present primarily as *cis*-isomers [72].

### 5.1. Prostatic Hyperplasia (PH)/Benign Prostatic Hyperplasia (BPH)

Prostatic hyperplasia (PH) develops in the majority of men along with the ageing process. Approximately 80% of men have PH at the age of 80. PH can result in urethral obstruction and lower urinary tract symptoms. The primary cause is the increased sensitivity of the prostate to dihydrotestosterone (DHT) [73]. Moreover, inflammatory processes may contribute to tissue injury and cytokines produced by inflammatory cells, which may stimulate local growth factor production and angiogenesis [74]. Drugs inhibiting DHT production, such as finasteride, have side effects associated with antiandrogenic properties. Consequently, new substances to combat PH are being tested.

Clinical trials are in compliance with the results from experimental studies discussed in part I of this review [1]. In a randomized, double-blinded, placebo-controlled trial, 90 patients received either placebo or LYC-Se-SeR for three months. Prostatic biopsies were conducted twice—at the beginning and at the end of the trial. After three months, there was a decrease in survivin and NAIP, while caspase-3 was significantly increased in BPH patients treated with SeR-Se-LYC, in comparison with the placebo group. This combination of compounds also reduced PSA and prostate-specific membrane antigen (PSMA) expression [75]. It has been hypothesized that SeR, LYC, and Se, administered together, can amplify their therapeutic efficacy also in terms of counteracting the inflammatory component of BPH. To verify this hypothesis, a comparison experiment was conducted. The anti-inflammatory activity of SeR, LYC, and Se on a proinflammatory phenotype in rat peritoneal macrophages stimulated with *Salmonella enteritidis* lipopolysaccharide (LPS) was compared to the effect observed in vivo in the prostate of rats with partial bladder outlet obstruction. As expected, LPS induced a proinflammatory phenotype in macrophages. LYC, Se, and SeR inhibited the inflammatory cascade, but it was the LYC-Se-SeR combination that caused a greater inhibitory effect on the expression of COX-2, 5-LOX, and iNOS. The LYC-Se-SeR association was effective in reducing the loss of IκB-α, NF-κB-binding activity, mRNA levels of TNF-α, malondialdehyde, and NO content [76]. In a multicenter study involving nine urological Italian centers between January 2009 and December 2010, prospectively collected data of two category of patients affected by BPH and/or PIN/ASAP (atypical small acinar proliferation) were analyzed. In both groups, the administration of LYC-Se-SeR (Profluss^®^) reduced the extension and grading of inflammation, with a significant decrease in inflammatory cells markers such as CD3, CD8, CD20, and CD68 [77].

It is worth mentioning that there have been some clinical trials evaluating treatment efficacy of other medical herbal preparations containing LYC. For instance, ProstateEZE Max, an orally dosed herbal preparation containing LYC, *Cucurbita pepo*, *Epilobium parviflorum*, *Pygeum africanum,* and *Serenoa repens* underwent a short-term phase II randomized double-blind placebo controlled clinical trial. Tested as a three-month intervention for the management of symptoms of medically diagnosed BPH in 57 otherwise healthy men aged 40–80 years, it reduced both daytime and night-time bladder emptying frequency. In the active group, a significant reduction in the median of the validated international prostate symptom score (IPSS) was observed [78].

### 5.2. Antioxidant Properties

LYC has some of the strongest antioxidant properties (in terms of quenching singlet oxygen) of carotenoids. Compared to BC, LYC has been reported to be twice as effective and 10-fold more active than α-tocopherol [79]. The effect of LYC supplementation on lipid peroxidation in plasma and on DNA oxidation in prostate tissue was studied in African American men aged 50–83 years old, recommended for prostate biopsy to detect cancer. In a randomized, double-blind, placebo-controlled phase II clinical investigation, 131 patients received oral doses of LYC or placebo for three weeks. Then, subjects underwent prostate needle biopsy for the diagnosis of BPH or PC, and further two extra biopsy specimens were obtained for measuring LYC concentration and DNA oxidation. A significant increase in prostate LYC levels in prostate tissue due to supplementation with a tomato extract was detected. Surprisingly, LYC supplementation did not significantly reduce the level of biomarkers of oxidative stress (malondialdehyde and 8-oxo-2’-deoxyguanosine) [80].

In an attempt to check the impact of LYC on soluble receptors for advanced glycation end products (sRAGE) in seminal and blood plasma, a randomized controlled trial was carried out. The study included 15 fertile volunteers and 13 normospermic male partners from infertile relationships. The 12-week administration of 20 mg of LYC or placebo was followed by a crossover and treatment for a further 12 weeks. sRAGE levels were determined using ELISA. LYC decreased sRAGE in seminal plasma, but not in blood plasma. A selective local uptake of LYC in the male reproductive tract, namely in the prostate, was suggested. Suppression of oxidative stress by LYC may explain, in part, the putative improvement in fertility reported after LYC treatment, as reactive oxygen species are known to oxidize sperm membrane lipids, proteins, and nucleic acids, leading to cellular dysfunction and subsequent male infertility [81].

## 6. Conclusions

This review presented insight into the recent findings about the influence of carotenoids and retinoids on prostate physiology and pathology, with special concern given to PC and prostatic hyperplasia. A strong potential of LYC in the prevention of PC is suggested, which seems to be increased in certain populations of men with TMPRSS2: ERG. For other carotenoids, sufficient evidence is lacking, and only under certain situations we may suspect an impact on PC (e.g., in smokers, BC lowers risk, while retinol increases it). Furthermore, regarding the treatment of PC, their roles remains still uncertain.

LYC, applied alone, or in combination with *Serenoa repens* and Se, counteracts prostate hyperplasia due to its proapoptotic, anti-inflammatory, antiandrogen, and antioxidant activity. Other properties of carotenoids, although being less frequently examined, are very promising, with a good example of astaxanthin and its apparently selective activity against PC cells, combined with a protective effect on normal prostate epithelial cells. However, as most studies were carried out with cell models, employing very high and nonphysiologic concentrations, it remains to be elucidated whether similar effects may occur in vivo.

The diversity of carotenoids and their influence on the human organism and prostate in particular still remains a source of fascinating, surprising findings. Undoubtedly, numerous discoveries in this field are awaiting us in the following years.

## Supplementary Materials

The following are available online at https://www.mdpi.com/2076-3921/10/2/319/s1, Figure S1: The flow chart summarizing the process of data extraction.
