# Supplementary material for: Recent Progress in Discovering the Role of Carotenoids and Metabolites in Prostatic Physiology and Pathology—A Review—Part II: Carotenoids in the Human Studies
Source: Antioxidants (Basel). 2021 Feb 20;10(2):319. doi: 10.3390/antiox10020319 (PMC7924028; doi:10.3390/antiox10020319)
Supplement: Supplementary file 1 [file antioxidants-10-00319-s001.pdf]

antioxidants

MDPI

Review

Recent Progress in Discovering the Role of Carotenoids and Metabolites in Prostatic Physiology and Pathology—A Review—Part II: Carotenoids in the Human Studies.

Joanna Dulińska-Litewka [1,*], Przemysław Hałubiec [1], Agnieszka Łazarczyk [1], Oskar Szafrański [1], Yoav Sharoni [2], James A. McCubrey [3], Bartosz Gąsiorkiewicz [1], Torsten Bohn [4]

[1] Chair of Medical Biochemistry Medical College, Jagiellonian University, 31-034 Cracow, Poland; przemyslawhalubiec@gmail.com (P.H.), agnieszka.lazarczyk@student.uj.edu.pl (A.Ł.), osk.sza2@gmail.com (O.S.), b.gasiorkiewicz@student.uj.edu.pl (B.G.).
[2] Department of Clinical Biochemistry, Faculty of Health Sciences, Ben-Gurion University of the Negev, P.O. Box 653, Beer Sheva, Israel; yoav@bgu.ac.il
[3] Department of Microbiology and Immunology, Brody Medical Sciences Building, East Carolina University, Greenville, NC 27834, USA; mccubreyj@ecu.edu
[4] Luxembourg Institute of Health, Population Health Department, Nutrition and Health Research Group 1 A-B, rue Thomas Edison, L-23 1445 Strassen, Luxembourg; Torsten.bohn@gmx.ch
* Correspondence: joanna.dulinska-litewka@uj.edu.pl; Tel.: +48-12-422-32-72

Supplementary

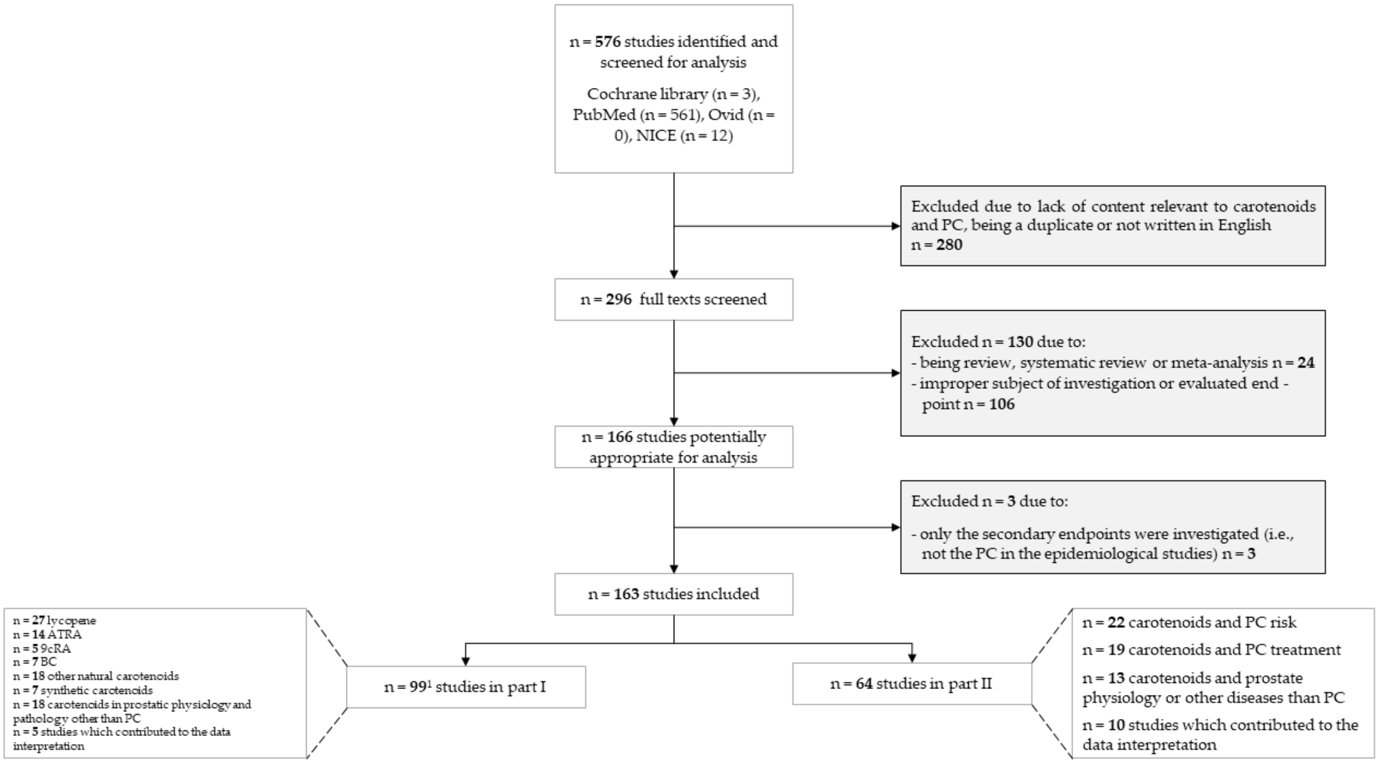

Figure 1. The flow chart summarizing the process of data extraction.
